# Inhibition of JAK-STAT Signaling Pathway Alleviates Age-Related Phenotypes in Tendon Stem/Progenitor Cells

**DOI:** 10.3389/fcell.2021.650250

**Published:** 2021-03-29

**Authors:** Minhao Chen, Longfei Xiao, Guangchun Dai, Panpan Lu, Yuanwei Zhang, Yingjuan Li, Ming Ni, Yunfeng Rui

**Affiliations:** ^1^Department of Orthopaedics, Zhongda Hospital, School of Medicine, Southeast University, Nanjing, China; ^2^Orthopaedic Trauma Institute (OTI), Southeast University, Nanjing, China; ^3^Trauma Center, Zhongda Hospital, School of Medicine, Southeast University, Nanjing, China; ^4^Department of Geriatrics, Zhongda Hospital, School of Medicine, Southeast University, Nanjing, China; ^5^Department of Orthopedics, The First Medical Center, Chinese PLA General Hospital, Beijing, China; ^6^China Orthopedic Regenerative Medicine Group, Hangzhou, China

**Keywords:** tendon-derived stem/progenitor cells, tendon aging, senescence, JAK-STAT signaling pathway, AG490

## Abstract

Diminished regeneration or healing capacity of tendon occurs during aging. It has been well demonstrated that tendon stem/progenitor cells (TSPCs) play a vital role in tendon maintenance and repair. Here, we identified an accumulation of senescent TSPCs in tendon tissue with aging. In aged TSPCs, the activity of JAK-STAT signaling pathway was increased. Besides, genetic knockdown of JAK2 or STAT3 significantly attenuated TSPC senescence in aged TSPCs. Pharmacological inhibition of JAK-STAT signaling pathway with AG490 similarly attenuated cellular senescence and senescence-associated secretory phenotype (SASP) of aged TSPCs. In addition, inhibition of JAK-STAT signaling pathway also restored the age-related dysfunctions of TSPCs, including self-renewal, migration, actin dynamics, and stemness. Together, our findings reveal the critical role of JAK-STAT signaling pathway in the regulation of TSPC aging and suggest an ideal therapeutic target for the age-related tendon disorders.

## Introduction

Tendon aging is characterized by time-dependent declines in structural, compositional, and functional properties (Zaseck et al., 2016; Marqueti et al., 2018). Aged tendon is more prone to tendon disorder, such as tendon tear, re-tear, and tendinopathy. Due to the limited endogenous repair capacity of aged tendon, the restoration of injured tendon is still a major clinical challenge. Currently, stem cell-based strategies to restore the original property of injured tendon are being investigated. Tendon tissue contains terminally differentiated tenocytes and a small resident tendon stem cell population, known as tendon stem/progenitor cells (TSPCs). TSPCs showed standard mesenchymal stem cell (MSC) characteristics, including self-renewal, clonogenicity, and multilineage differentiation potential; TSPCs also express classical MSC surface antigens and tendon lineage genes (Bi et al., 2007; Lui and Wong, 2019). TSPCs are essential for effective repair, regeneration, and maintenance of tendon. Previous studies have demonstrated that TSPCs features alter during tendon aging; aged TSPCs premature entry into senescence and exhibit profound self-renewal, migration, and tenogenic differentiation deficits (Kohler et al., 2013; Yin et al., 2020). These age-related changes of TSPCs led to impaired tendon healing and regeneration capacity (Dai et al., 2019; Li et al., 2019). Although the link between TSPCs and tendon aging has been well recognized, the specific mechanisms underlying age-related TSPC dysfunction remain incompletely clarified.

The Janus kinase-signal transducer and activator of transcription (JAK-STAT) signaling pathway is a cytokine-stimulated signal transduction pathway and involves in various biological processes, including differentiation, immune regulation, proliferation, and hematopoiesis (Herrera and Bach, 2019; Salas et al., 2020). Studies have also demonstrated that JAK-STAT signaling pathway plays a vital role in stem cell aging. In satellite cells, increased JAK-STAT signaling pathway impairs muscle regeneration during aging, and JAK-STAT signaling inhibition enhances satellite cell expansion, muscle repair, and functional performance (Price et al., 2014). Xu et al. (2015b) reported that inhibition of JAK-STAT signaling pathway attenuates the senescence-associated secretory phenotype (SASP) in aged preadipocytes and umbilical vein endothelial cells; JAK-STAT inhibition also decreases age-related inflammation and frailty. Doles et al. (2012) showed that increased JAK-STAT signaling inhibits hair follicle stem cell function in aged mice. In addition, our previous study has demonstrated that JAK-STAT signaling pathway is activated in TSPCs of Achilles tendon from aged rats (Chen et al., 2020). Therefore, these findings prompted us to investigate the specific role of the JAK-STAT signaling pathway in TSPC aging and age-related dysfunction.

In the present study, we confirm that senescent TSPCs accumulate in tendon tissue with aging. We showed that increased JAK-STAT signaling induces TSPC senescence. Inhibition of JAK-STAT signaling pathway attenuates cell senescence and SASP in aged TSPCs. Moreover, JAK-STAT inhibitor restored the age-related dysfunction of self-renewal, migration, and stemness. Our findings provide insights into the possible contribution of senescent TSPCs to age-related tendon disorder. JAK-STAT signaling pathway could be an ideal therapeutic target to antagonize tendon aging.

## Materials and Methods

### TSPC Isolation and Culture

For TSPC isolation and culture, 4- and 20-month-old male Sprague-Dawley rats were used (*n* = 10). Rat TSPCs were isolated from young and aged Achilles tendons as previously described (Rui et al., 2010). All experimental procedures were approved by the Animal Research Ethics Committee of Southeast University. Briefly, the Achilles tendons were minced and digested with type I collagenase (3 mg/ml; Sigma-Aldrich), then gently passed through 70 μm cell strainers (Becton Dickinson) to obtain single-cell suspensions. The cells were cultured in Dulbecco’s modified essential medium (DMEM), supplemented with 10% fetal bovine serum, and 1% penicillin-streptomycin (all from Gibco) at 5% CO_2_ (37°C). Fresh culture medium was changed every 3 days. Cells from passages 2–6 were used for the experiments.

### Cell Transfection

JAK2-siRNA and STAT3-siRNA were designed and synthetized from GenePharma (Shanghai, China). The sequence of JAK2-siRNA was as follows: GCACAUCAGAAUGGUGAUATT (sense: 5′-3′), UAUCACCAUUCUGAUGUGCTT (anti-sense: 5′-3′). The sequence of STAT3-siRNA was as follows: GGCAUUCGGAAAGUAUUGUTT (sense: 5′-3′), ACAAUACUUUCCGAAUGCCTT (anti-sense: 5′-3′). When cells reached 50% confluence, they were transfected with siRNAs using the jetPRIME transfection reagent (Polyplus) according to the manufacturer’s protocol. At 48 h after transfection, the cells were harvested and used for subsequent assays.

### RNA Sequencing

The RNA sequencing was performed by Beijing CapitalBio Corporation (Beijing, China) as previously described (Chen et al., 2020). The libraries were generated using NEBNext Ultra RNA Library Prep Kit for Illumina and sequenced on the Illumina HiSeq 2500 platform. For data analysis, genes with fold change ≥ 2 and *P*-value < 0.05 were recognized as to be significantly differentially expressed genes. Clustering analysis and heat map construction were performed with Cluster 3.0 software. For gene set enrichment analysis (GSEA, Broad Institute), the significant difference was verified using the normalized enrichment score (NES) and false discovery rate (FDR).

### Quantitative RT-PCR

Total RNA of the TSPCs was extracted using MiniBEST universal RNA extraction kit (Takara) according to the manufacturer’s instructions. The First-Strand cDNA kit (Promega) was used to synthesize cDNA from mRNA. Each PCR reaction was conducted in a 20-μl reaction mixture containing 1 μl of total cDNA, Power SYBR Green PCR Master Mix (Invitrogen), and specific primers. Quantitative RT-PCR was performed using the ABI Step-One Plus system. The cycling parameters were as follows: 95°C for 10 min, followed by 45 cycles at 95°C for 20 s, optimal annealing temperature for 20 s, 72°C for 30 s, and finally 60–95°C in increments of 0.1°C/s. The relative expression of each target gene was normalized with β-actin gene. Relative gene expression fold change was calculated with 2^–ΔΔCt^ method. The primers used for PCR are listed in Supplementary Table 1.

### Western Blotting

Total protein of the TSPCs was extracted in cold RIPA lysis buffer (Keygen biotech), and the protein concentration was measured using BCA protein assay kit (Thermo Scientific). A total of 30 μg of protein was electrophoresed on SDS-PAGE. After electrotransfer onto PVDF membrane (Millipore), the membranes were blocked with PBST containing 5% non-fat dry milk for 1 h at room temperature. The membranes were then incubated with primary antibody against JAK2 (Proteintech), p-JAK2 (Abcam), STAT3 (Proteintech), p-STAT3 (Abcam), p16^INK4A^ (Beyotime Biotechnology), cyclin D1 (Bioworld), cyclin B1 (Proteintech), and GAPDH (Proteintech) at 4°C overnight. After washing with PBST, the membranes were incubated with secondary antibody and immunoreactive bands were visualized with ECL reagents (Keygen biotech).

### β-Galactosidase Staining

β-Galactosidase staining was performed with the senescence-associated β-galactosidase staining kit (Sigma). TSPCs were plated in 12-well plates and incubated for 48 h; cells were then washed with PBS and fixed in 4% paraformaldehyde for 15 min. Next, cells were incubated with staining mixture for 16 h at 37°C. The percentage of positive cells was calculated by counting at least 300 cells in six microscopic fields. Images were captured using an Olympus inverted phase contrast microscope.

### Cell Cycle Analysis

Analysis of cell cycle distribution was assessed using the cell cycle detection kit (Keygen Biotech). The TSPCs were cultured for 24 h before being trypsinized and detached. Cells were then fixed in 70% ethanol overnight at 4°C. After being washed, the ethanol-fixed cells were incubated with RNase A and propidium iodine for 30 min. The cell cycle distributions were analyzed on flow cytometry (Becton-Dickinson) using Cell Quest software.

### Colony Forming Unit Assays

Tendon stem/progenitor cells were seeded at a density of 2, 5, and 10 cells/cm^2^ in six-well plates respectively and cultured for 10 days to form colonies. Cells were fixed in 4% paraformaldehyde for 15 min and stained with 0.5% crystal violet for counting the number of colonies. Colony forming unit (CFU) efficiency was calculated as follows: counted colonies/number of inoculated cells × 100.

### EdU Detection

For EdU detection, the EdU Cell Proliferation Kit with Alexa Fluor 594 (Beyotime Biotechnology) was used according to the manufacturer’s protocol. Briefly, cultured TSPCs were incubated with 10 μM EdU for 5 h, then fixed in 4% paraformaldehyde for 15 min at room temperature. After being washed, cells were permeabilized with 0.3% Triton X-100 for 10 min, then cells were stained with click additive solution for 30 min. Immunofluorescence was visualized using a Nikon Ts2R fluorescence microscope.

### CCK-8 Assay

Cell proliferation was measured using the Cell Counting Kit-8 (CCK-8, Keygen biotech) assay. TSPCs were seeded at a density of 3,000 cells/well in 96-well plates and cultured for 0, 24, 48, and 72 h, respectively. The cells were then treated with 10 μl of CCK-8 reagent and incubated for 2 h at 37°C. The absorbance value of each well was measured at 450 nm.

### Population Doubling Time Assay

Population doubling time (PDT) was calculated using the formula log_2_ [*N*_c_/*N*_0_], where *N*_c_ is the total cell number at confluence and *N*_0_ is the number of inoculated cells.

### TSPCs Migration Assay

A scratch assay was used to measure TSPC migration. TSPCs were seeded in six-well plates and grown to confluence. The cell layer was then scratched with a sterile pipette tip and incubated for 16 h. Images were captured using an Olympus inverted phase contrast microscope. Cell velocity was quantified by scratch length and scratch bridging time.

### Investigation of Actin Dynamics

For actin dynamics analysis, TSPCs were seeded in six-well plates and incubated for 48 h. Cells were then treated with 0.4 μM latrunculin A (Sigma Aldrich) for 0, 15, 30, and 60 min, respectively. Next, cells were fixed in 4% paraformaldehyde for 15 min at room temperature. After being washed, cells were permeabilized with 0.1% Triton X-100 for 10 min, then cells were stained with Alexa Flour 546 phalloidin (Thermo Scientific) for 1 h at room temperature. Immunofluorescence was visualized using a Nikon Ts2R fluorescence microscope.

### Statistical Analysis

All data are plotted as mean ± SEM. Unpaired *t*-test was used for comparisons between two groups, and one-way analysis of variance (ANOVA) followed by Tukey’s *post hoc* test was used for multiple comparisons. *P* < 0.05 were considered to be statistically significant. Each experiment had three replicates per condition, and the experiment was performed three times.

## Results

### TSPCs Exhibit a Senescent Phenotype With Advancing Age

**FIGURE 1:**
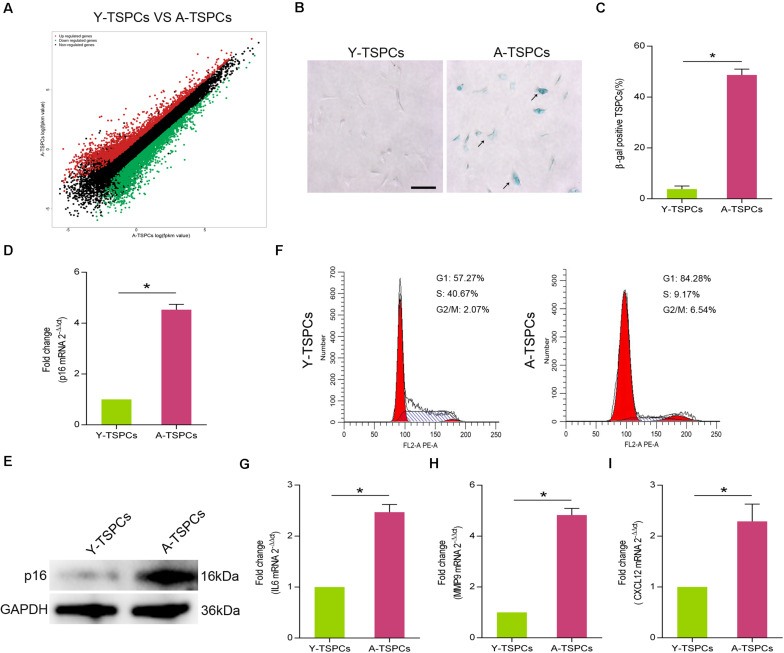
The TSPCs from aged Achilles tendon showed senescence characteristics. **(A)** RNA-seq analysis of young and aged TSPCs. Scatter plot represents fpkm values for genes expressed in young and aged TSPCs. Red dots show the upregulated and green dots show the downregulated genes (fold change ≥ 2). (**B**,**C**) β-gal staining for the senescent cells in young and aged TSPCs. Scale bars: 100 μm. **(D)** Relative mRNA levels of p16^INK4A^ in young and aged TSPCs were investigated by qRT-PCR. **(E)** Western blotting for the p16^INK4A^ protein levels in young and aged TSPCs. **(F)** The cell cycle distribution of TSPCs was measured by flow cytometry. (**G**–**I**) qRT-PCR for SASP gene (IL6, MMP9, and CXCL12) expressions in young and aged TSPCs. *P < 0.05 compared with young group.

To characterize the senescent phenotype in TSPCs, we isolated TSPCs from Achilles tendon of 4-month-old (Y-TSPC) and 20-month-old (A-TSPC) Sprague-Dawley rats. We first performed RNA sequencing on young and aged TSPCs; we found substantial changes in transcriptome. The results showed 2,142 upregulated mRNAs and 2,455 downregulated mRNAs in aged TSPCs (Figure 1A). In addition, we observed increased in the number of β-galactosidase (β-gal)-positive senescent cells in aged TSPCs (Figures 1B,C). This was coupled with a significant upregulation of senescence-associated marker p16^INK4A^ in aged TSPCs (Figures 1D,E). Cell cycle arrest is an important indication of cellular senescence (Gorgoulis et al., 2019); we next investigated cell cycle phase distribution of TSPCs. The results revealed an accumulation of aged TSPCs in G1 phase, with a reduced number of cells in the S and G2/M phases (Figure 1F). Moreover, we investigated the expressions of some representative SASP genes, including interleukin (IL)6, matrix metalloproteinase (MMP)9, and C-X-C motif chemokine ligand 12 (CXCL12), and the results showed that these SASP gene levels were highly increased in aged TSPCs (Figures 1G–I). Collectively, our findings suggested that the aged tendon contains an increased proportion of senescent TSPCs, which could induce tissular and cellular dysfunction.

### JAK-STAT Signaling Pathway Is Activated in Aged TSPCs

**FIGURE 2:**
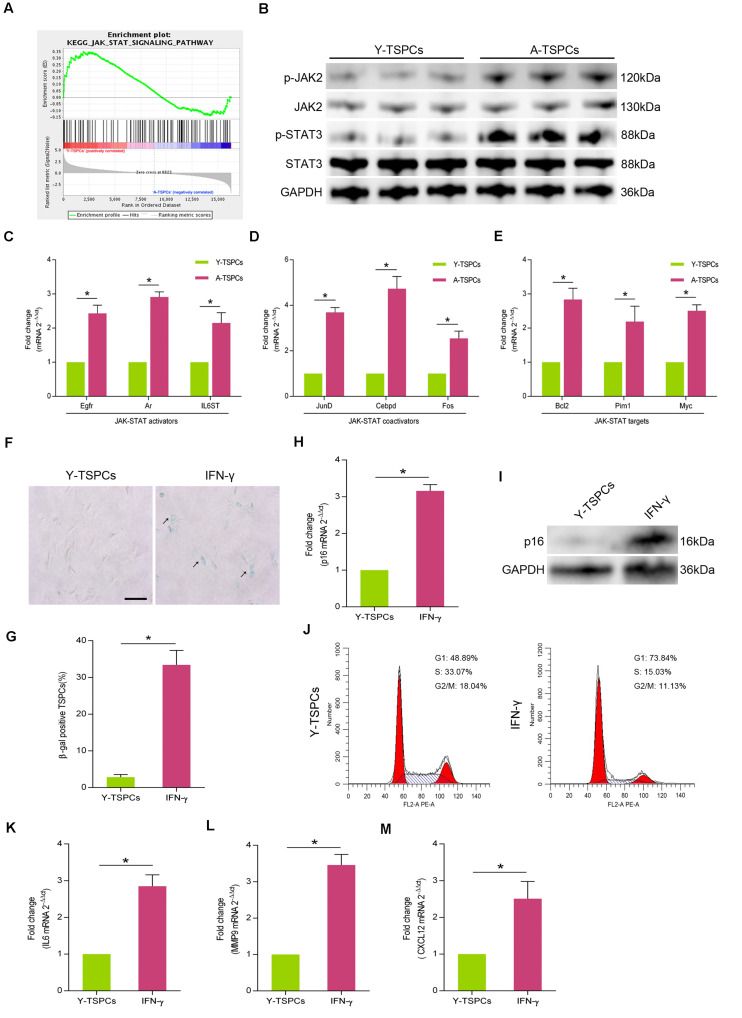
The activity of JAK-STAT signaling pathway is increased with age. **(A)** GSEA plots showed a gene set related to JAK-STAT pathway. **(B)** Western blotting for the p-JAK2, JAK2, p-STAT3, and STAT3 protein levels in young and aged TSPCs. (**C**–**E**) Relative mRNA levels of JAK-STAT activators (Egfr, Ar, and IL6ST), JAK-STAT coactivators (JunD, Cebpd, and Fos), and JAK-STAT targets (Bcl2, Pim1, and Myc) in young and aged TSPCs were validated by qRT-PCR. (**F**,**G**) β-gal staining for the senescent cells in young TSPCs upon IFN-γ (100 ng/ml, 24 h) treatment. Scale bars: 100 μm. (**H**) qRT-PCR for the p16^INK4A^ mRNA levels in young TSPCs upon IFN-γ treatment for 8 h. (**I**) Western blotting for the p16^INK4A^ protein levels in young TSPCs after treatment with IFN-γ (100 ng/ml, 8 h). (**J**) Flow cytometry analysis was used to measure the cell cycle distribution of young TSPCs after IFN-γ treatment for 48 h. (**K**–**M**) qRT-PCR for SASP genes (IL6, MMP9, and CXCL12) expressions in young TSPCs upon IFN-γ treatment for 8 h. *P < 0.05 compared with the young group.

We analyzed gene sets using the Kyoto encyclopedia of genes and genomes (KEGG) suite in Gene set enrichment analysis (GSEA) to identify enriched signaling pathways in young and aged TSPCs. The GSEA KEGG analysis showed a significant decrease in enrichment of genes associated with JAK-STAT signaling pathway in aged TSPCs (Figure 2A). To study the activity of JAK-STAT signaling pathway in aged TSPCs, we investigated the expressions of p-JAK2 and p-STAT3 in young and aged TSPCs, and the result showed that the protein levels of p-JAK2 and p-STAT3 were notably increased in aged TSPCs (Figure 2B). Moreover, we examined the expressions of some representative genes involved in JAK-STAT signaling pathway, and we found significant higher levels of JAK-STAT activators (Egfr, Ar, and IL6ST), JAK-STAT coactivators (JunD, Cebpd, and Fos), and JAK-STAT targets (Bcl2, Pim1, and Myc) in TSPCs from aged rat relative to young rat (Figures 2C–E). We next treated young TSPCs with interferon-γ (IFN-γ), an activating cytokine of JAK-STAT signaling pathway. The results showed that IFN-γ treatment increased the percentage of β-gal-positive senescent cells (Figures 2F,G), as well as the expression of p16^INK4A^ in young TSPCs (Figures 2H,I). Cell cycle analysis revealed that IFN-γ treatment induced G1 arrest in young TSPCs (Figure 2J). Moreover, IFN-γ treatment also increased the mRNA levels of IL6, MMP9, and CXCL12 in young TSPCs (Figures 2K–M). These results indicated that the activity of JAK-STAT signaling pathway is increased with age, which might be associated with the senescence of TSPCs.

### Inhibition of JAK-STAT Signaling Pathway Attenuates TSPC Senescence

**FIGURE 3:**
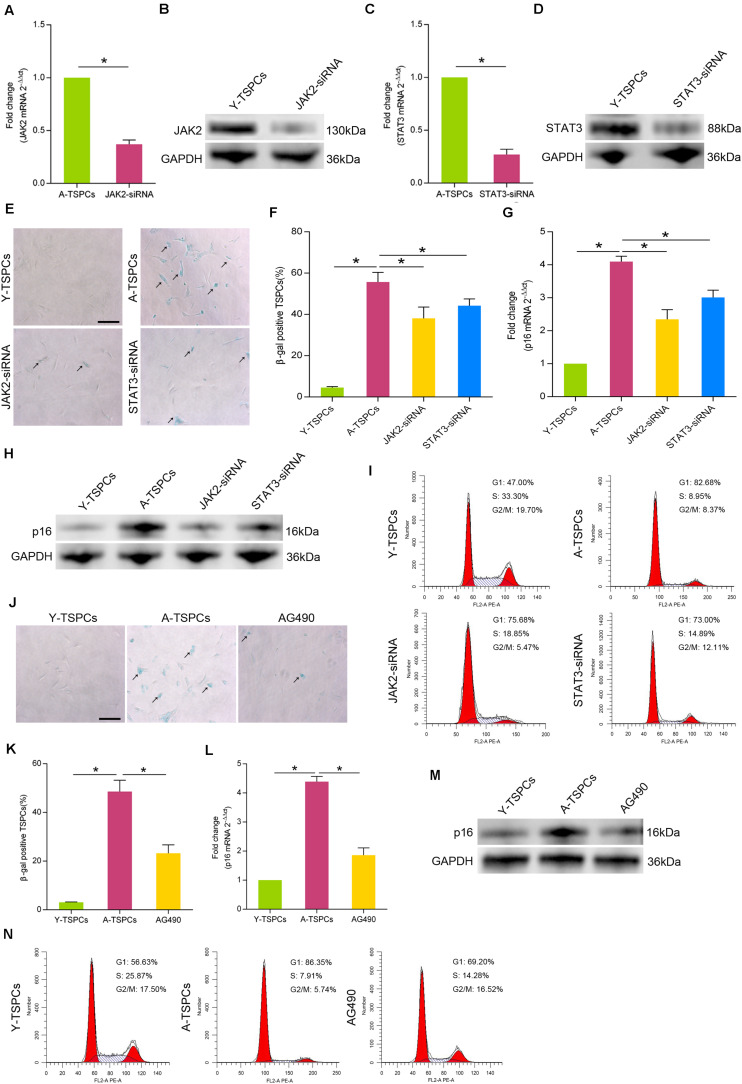
Inhibition of JAK-STAT signaling pathway inhibits cellular senescence. (**A**,**B**) The transfection efficiency of JAK2-siRNA was explored by qRT-PCR and Western blotting in aged TSPCs. (**C**,**D**) The expression of STAT3 mRNA and protein was measured by qRT-PCR and Western blotting in aged TSPCs after treatment with STAT3-siRNA. (**E**,**F**) β-gal staining for the senescent cells in aged TSPCs upon JAK2-siRNA or STAT3-siRNA treatment. Scale bars: 100 μm. (**G**,**H**) The expression of p16^INK4A^ mRNA and protein was measured by qRT-PCR and Western blotting. (**I**) The cell cycle distribution of TSPCs was measured by flow cytometry after JAK2-siRNA/STAT3-siRNA treatment. (**J**,**K**) β-gal staining for the senescent cells in aged TSPCs after treatment with AG490 treatment (100 μM, 24 h). Scale bars: 100 μm. (**L**,**M**) The p16^INK4A^ level was investigated by qRT-PCR and Western blotting in young, aged, and aged AG490-treated TSPCs. (**N**) Cell cycle analysis of aged TSPCs after AG490 treatment for 48 h. *P < 0.05 compared with the young or aged group.

To investigate the specific role of JAK-STAT signaling pathway in TSPC senescence, we treated aged TSPCs with JAK2-siRNA or STAT3-siRNA. The transfection efficiency was detected by Western blotting and qRT-PCR (Figures 3A–D). Our results showed that treatment with JAK2-siRNA or STAT3-siRNA significantly reduced the β-gal-positive senescent cells in aged TSPCs (Figures 3E,F). Besides, JAK2-siRNA or STAT3-siRNA treatment also decreased the p16^INK4A^ level of aged TSPCs (Figures 3G,H). Cell cycle analysis revealed that the accumulation of aged TSPCs at G1 phase was rescued after JAK2 or STAT3 knockdown (Figure 3I). In addition, we also treated aged TSPCs with the JAK-STAT signaling pathway inhibitor AG490. Notably, AG490 treatment showed an attenuated senescent phenotype, as indicated by reduced β-gal-positive senescent cells (Figures 3J,K) and p16^INK4A^ level (Figures 3L,M), as well as the accumulation of G1 phase (Figure 3N). Together, these findings further indicated that the JAK-STAT signaling pathway plays an essential role in regulating TSPC senescence.

### JAK-STAT Signaling Pathway Inhibitor Suppresses the SASP in Aged TSPCs

**FIGURE 4:**
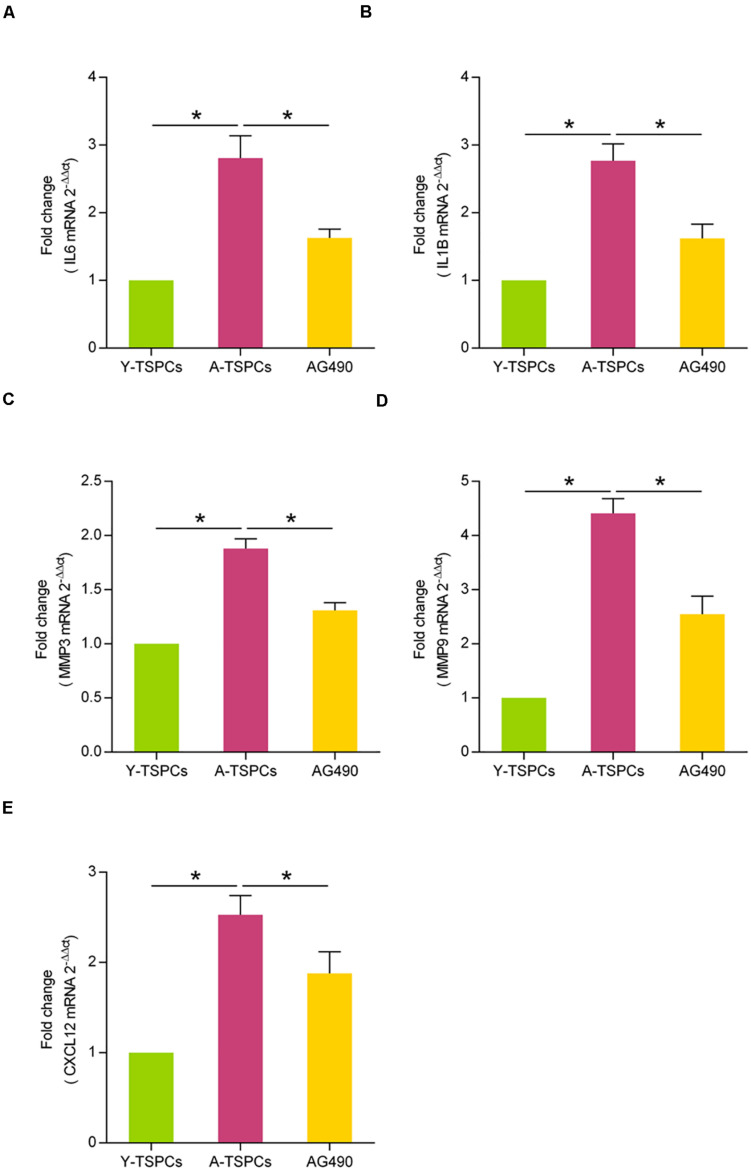
AG490 inhibits the SASP expression in aged TSPCs. (**A–E**) Relative mRNA levels of representative SASP genes (IL6, IL1B, MMP3, MMP9, and CXCL12) in young, aged, and aged AG490-treated TSPCs were investigated by qRT-PCR. *P < 0.05 compared with the young or aged group.

Given that the inhibition of JAK-STAT signaling pathway could attenuate cellular senescence in aged TSPCs, we examined whether inhibiting the JAK-STAT pathway blunts the SASP gene expressions. Using qRT-PCR, we investigated the expressions of some representative SASP genes, including IL6, IL1B, MMP3, MMP9, and CXCL12. The results showed that these SASP gene expressions were significantly increased in aged TSPCs (Figures 4A–E), which suggested that TSPCs exhibit a SASP upon becoming senescent. Meanwhile, AG490 treatment reduced the SASP gene levels of aged TSPCs (Figures 4A–E). Collectively, our findings indicated that the capacity of JAK-STAT signaling pathway inhibitor suppresses the SASP in aged TSPCs.

### AG490 Restores the Self-Renewal Deficit of Aged TSPCs

**FIGURE 5:**
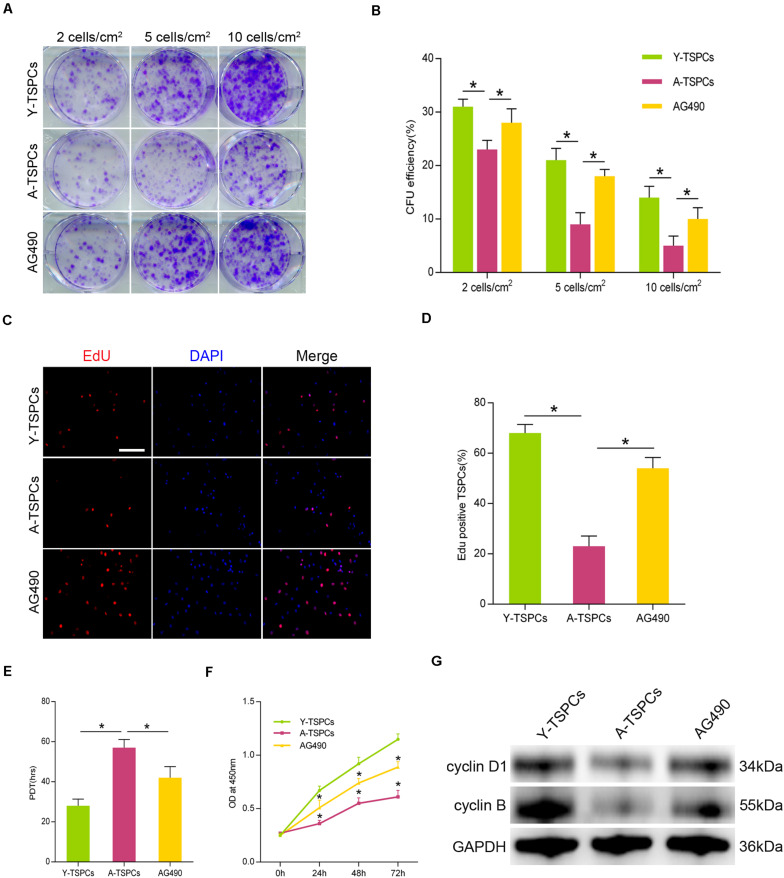
Inhibition of JAK-STAT signaling pathway increases aged TSPC self-renewal. (**A**) Colony forming unit (CFU) assay showed multiple TSPC colonies stained with crystal violet at three densities. (**B**) Colony forming efficiency of young, aged, and aged AG490-treated TSPCs. (**C**,**D**) Representative images for EdU staining and EdU incorporation was quantified as EdU^+^ cells/total cells. Scale bar: 50 μm. (**E**,**F**) Cell proliferation was investigated by population doubling time (PDT) assay and CCK-8 assay. (**G**) The protein levels of cyclin D1 and cyclin B were analyzed by Western blotting. *P < 0.05 compared with the young or aged group.

To investigate the role of JAK-STAT signaling pathway in TSPC self-renewal, we treated aged TSPCs with AG490. The results showed that AG490 significantly increased the colony number and CFU efficiency in the aged TSPCs compared with the young TSPCs (Figures 5A,B). We next measured the percentage of proliferating cells after incubation with 5-ethynyl-2′-deoxyuridine (EdU), and AG490 treatment increased the percentage of EdU-positive senescent cells in aged TSPCs (Figures 5C,D). Moreover, the PDT and CCK-8 assay also demonstrated the ability of AG490 to enhance the proliferative potential of aged TSPCs (Figures 5E,F). To further explore the changes at molecular level, we investigated the expressions of cell proliferation-related proteins in TSPCs, and we found that the expressions of cyclin D1 and cyclin B were significantly reduced in aged TSPCs, while the reduced levels of these proteins were rescued after AG490 treatment (Figure 5G). Together, our results suggested that inhibition of JAK-STAT signaling pathway improves the self-renewal ability of aged TSPCs.

### Inhibition of JAK-STAT Signaling Pathway Promotes Migration and Actin Dynamics of Aged TSPCs

**FIGURE 6:**
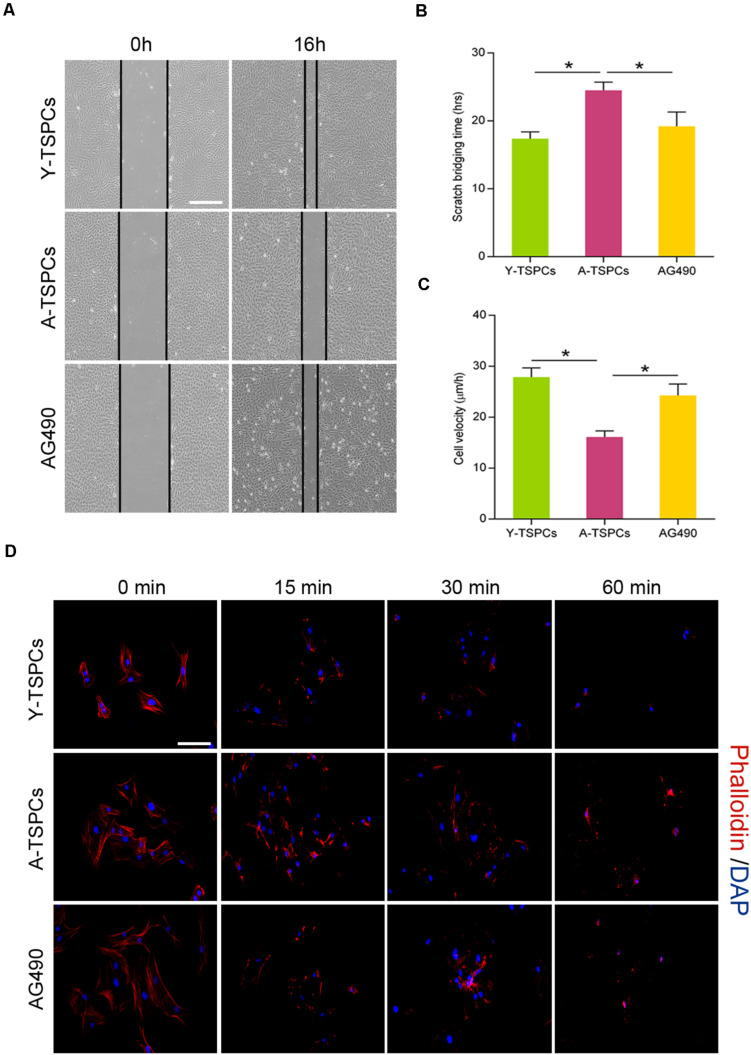
AG490 enhances aged TSPC migration and actin dynamics. (**A**) Representative scratch assay of young, aged, and aged AG490-treated TSPCs. Scale bar: 200 μm. (**B**,**C**) TSPC migration rate was calculated by scratch bridging time and cell velocity. (**D**) Phalloidin staining for F-actin in young, aged, and aged AG490-treated TSPCs at each time point after latrunculin A treatment. Scale bar: 20 μm. *P < 0.05 compared with the young or aged group.

Tendon stem/progenitor cell migration is required for tendon-healing process. We tested if JAK-STAT signaling pathway suppression improves the migration deficit of aged TSPCs. We performed a scratch assay to mimic wound closure, and the result showed that the migration speed and distance of aged TSPCs were slower than young TSPCs, while AG490 restores the migration deficit of aged TSPCs (Figures 6A–C). Since the turnover rate of the actin cytoskeleton is important for cell migration, we performed phalloidin staining to compare the actin dynamics by treating TSPCs with latrunculin A in a time-dependent manner. We observed a slower rate of actin turnover in aged TSPCs compared with the young TSPCs, and AG490 significantly reduced the F-actin content in aged TSPCs, which suggested that AG490 increases actin turnover of aged TSPCs (Figure 6D). Collectively, these results indicated that inhibition of JAK-STAT signaling pathway restores the migration deficit and improves actin dynamics of aged TSPCs.

### AG490 Promotes Stemness and Tendon-Related Marker Expressions of Aged TSPCs

**FIGURE 7:**
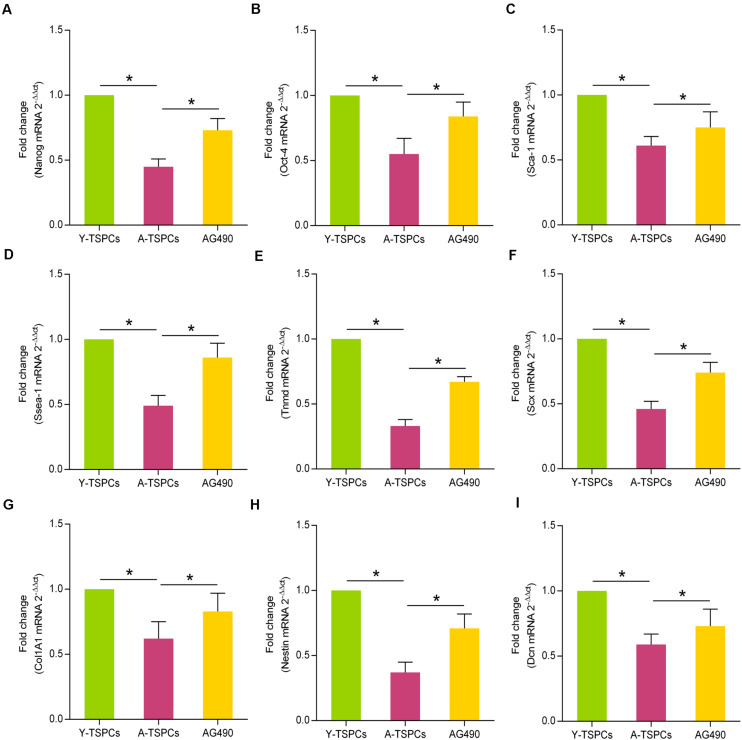
AG490 increases the expressions of stem cell markers and tendon-related genes in aged TSPCs. (**A**–**D**) Relative mRNA levels of stem cell markers (Nanog, Oct-4, Sca-1, and Ssea-1) in young, aged, and aged AG490-treated TSPCs were investigated by qRT-PCR. (**E**–**I**) qRT-PCR for the tendon-related genes (Tnmd, Scx, Col1A1, Nestin, and Dcn) upon AG490 treatment. *P < 0.05 compared with the young or aged group.

To study the role of JAK-STAT signaling pathway in the stemness of TSPCs, we investigated the expressions of some representative stem cell markers, including Nanog, Oct-4, Sca-1, and Ssea-1. The results revealed that these markers were reduced in aged TSPCs, which suggested that aging suppresses the stemness of TSPCs, and AG490 treatment reverses the inhibitory effect of aging on stemness in aged TSPCs (Figures 7A–D). Given the ability of AG490 to promote stemness of aged TSPCs, we next investigated the role of JAK-STAT signaling pathway in tenogenic differentiation of TSPCs. We examined the mRNA levels of the key tendon-related marker, including Tnmd, Scx, Col1A1, Nestin, and Dcn, and these markers were significantly downregulated in aged TSPCs, which suggested a lower tenogenic differentiation capacity of aged TSPCs. In addition, AG490 treatment could increase the expressions of the tendon-related markers in aged TSPCs (Figures 7E–I). Together, these findings indicated a critical role of JAK-STAT signaling pathway in stemness and tenogenic differentiation of TSPCs.

## Discussion

Tendon stem/progenitor cell function declines with age. Our study showed that TSPCs isolated from aged tendon exhibit a senescent phenotype, as evidenced by increased expression of senescence-associated marker (p16^INK4A^, β-gal), cell cycle arrest, and SASP. Cellular senescence is a cell state related to various physiological processes and a series of age-related diseases (Gorgoulis et al., 2019). The accumulation of senescent cells in aged TSPCs not only offer new insights into the biological mechanisms of TSPCs aging but also indicate that age-related TSPC dysfunction could be linked to the pathological changes of aged tendon. In the present study, we demonstrated that activation of the JAK-STAT signaling pathway is closely correlated with TSPC senescence. The JAK-STAT signaling pathway is an important intracellular pathway and has been shown to play a vital role in aging (Shen-Orr et al., 2016; Lenhart et al., 2019). As JAK-STAT signaling pathway is frequently activated during the aging process, JAK inhibitors are being widely used to alleviate age-related dysfunction (Tierney et al., 2014; Qin et al., 2016; Verstovsek et al., 2017). These findings raise the possibility that inhibition of the JAK-STAT signaling is a potentially promising strategy for antagonizing TSPC aging. Consistent with this possibility, we treated aged TSPCs with inhibitors of the JAK-STAT signaling pathway JAK2-siRNA, STAT3-siRNA, and AG490; notably, treatment with these inhibitors significantly attenuated TSPC senescence, highlighting the role of JAK-STAT signaling pathway in TSPCs. In contrary, treatment of young TSPCs with activating cytokine of JAK-STAT signaling pathway IFN-γ promoted premature senescence of young TSPCs. Collectively, the role of JAK-STAT signaling pathway in promoting senescence may represent a new mechanism in TSPC aging.

A hallmark of aging is chronic sterile inflammation, and increased pro-inflammatory cytokines and cytokines are closely associated with age-related diseases (Lopez-Otin et al., 2013; Tchkonia et al., 2013; Xu et al., 2015b). Studies have demonstrated that inflammation is also associated with tendon aging, and chronic, low-grade inflammation can damage tendon structure (Gaida et al., 2009; Dakin et al., 2012). Senescent cells exhibit a SASP, and the SASP contains large amounts of pro-inflammatory cytokines, matrix metalloproteinases, and chemokines and contributes to persistent chronic inflammation (Coppe et al., 2010a,b; Franceschi and Campisi, 2014). Therefore, the harmful and pro-aging effects of senescent cells are partly due to the SASP. Previous studies have reported that preadipocytes, endothelial cells, and omental adipose tissue cells develop a SASP with aging (Xu et al., 2015a, 2018). In the present study, a robust pattern of SASP induction is shown in aged TSPCs, which suggests the possibility that senescent TSPCs promotes tendon aging. Studies have demonstrated that the JAK-STAT signaling pathway mediates numerous pro-inflammatory cytokine expressions (Yu et al., 2009; Xu et al., 2015b; Salas et al., 2020). Inhibition of JAK-STAT signaling pathway seems to be an ideal choice for suppressing SASP; consistent with this possibility, we also showed that JAK inhibitor AG490 decreased the SASP in aged TSPCs. Thus, our results suggested that the JAK-STAT signaling pathway plays a vital role in the SASP and could be an ideal target for alleviating SASP during tendon aging.

Self-renewal ability is a critical property of stem cell to regulate tissue regeneration and homeostasis (Alberton et al., 2015). TSPCs self-renew to maintain a pool of healthy stem cells (Zhang and Wang, 2010). TSPCs exhibit a reduced self-renewal during tendon aging, which may be the key mechanism for ineffective self-renewal and impaired tendon regeneration capacity (Kohler et al., 2013; Zhang and Wang, 2015; Chen et al., 2020). Consistent with previous studies, our results also showed an attenuated self-renewal ability in aged TSPCs, as evidenced by decreased clonogenic potential and proliferation. It has been reported that JAK-STAT signaling pathway plays an important role in stem cell self-renewal, and appropriate levels of JAK-STAT signaling pathway are required for stem cell maintenance and self-renewal (Tulina and Matunis, 2001; Flaherty et al., 2010). In the present study, we reported that inhibition of JAK-STAT signaling pathway significantly enhanced clonogenic capacity and proliferative rate of aged TSPCs, supporting a potential therapeutic role of JAK-STAT signaling pathway in self-renewal of aged TSPCs.

Studies have shown that aged TSPCs exhibit marked reduction in cell migration and actin dynamics of TSPC during tendon aging (Kohler et al., 2013; Popov et al., 2015). Here, we demonstrated that JAK-STAT signaling pathway inhibitor significantly promoted migration and actin dynamics of aged TSPCs. These findings are in line with a benefit effect of JAK-STAT signaling pathway inhibitor in cell migration and actin dynamics as several studies reported (Tu et al., 2012; Ji et al., 2017). Migration is an important repair-mediating TSPC function, enabling TSPCs to move toward and repair damaged tendon tissues (Dekoninck and Blanpain, 2019). Thus, by improving migration deficit in aged TSPCs, inhibition of JAK-STAT signaling pathway may provide a more effective treatment strategy for age-related tendon disorders. Besides, given that the actin cytoskeleton and its dynamics are vital for cell migration (Rottner and Stradal, 2011), the lower rate of actin turnover offers a possible explanation for the migration deficit in aged TSPCs. Therefore, the enhanced migratory capacity of aged TSPCs may be associated with the improved actin dynamics after JAK-STAT signaling pathway inhibitor treatment.

Differentiation capacity is another key property of TSPCs regulating tendon regeneration and homeostasis (Bi et al., 2007). TSPCs express pluripotency-related factors (Shi et al., 2017; Liu et al., 2018), but their expression decreased with age (Zhang and Wang, 2015). In the present study, we showed that the expressions of Nanog, Oct-4, Sca-1, and Ssea-1 were reduced in aged TSPCs. Nanog and Ssea-1 are involved in the maintenance of stemness in undifferentiated embryonic stem cells (Cui et al., 2004; Pan and Thomson, 2007). Oct-4 is essential for maintaining pluripotency in stem cells (Pesce and Scholer, 2001). Sca-1 is widely recognized as a stem cell marker (Raghuvanshi et al., 2010). Thus, the reduced expressions of these stem cell markers in aged TSPCs observed here indicated that aging suppresses the stemness of TSPCs. In addition, studies have demonstrated that JAK-STAT signaling pathway plays an essential role in stem cell differentiation (Lin et al., 2010; Tanaka et al., 2018). Here, we found that inhibition of JAK-STAT signaling pathway increased these stem cell markers in aged TSPCs, which suggested a vital role of JAK-STAT signaling pathway in TSPC stemness. We next used several tendon-related markers (Tnmd, Scx, Col1A1, Nestin, and Dcn) to investigate the potential role of JAK-STAT signaling pathway in the tenogenic differentiation ability of TSPCs. These tendon-related markers have been shown to be reduced in aged TSPCs, which suggested an impaired tenogenic differentiation ability of aged TSPCs (Zhang et al., 2016; Han et al., 2017). Moreover, inhibition of JAK-STAT signaling pathway restored the age-related reduction of these tendon-related markers in aged TSPCs. Therefore, our results also showed a critical role of JAK-STAT signaling pathway in tenogenic differentiation of TSPCs.

## Conclusion

In summary, our study suggested that aberrant JAK-STAT signaling pathway is an important contributor to TSPC senescence during tendon aging. Inhibition of JAK-STAT signaling pathway attenuated cellular senescence and SASP in aged TSPCs. In addition, inhibition of JAK-STAT signaling pathway also restored the age-related reduction of self-renewal, migration, actin dynamics, and stemness in TSPCs. Our findings suggested a promising therapeutic target for age-related tendon disorders.

## Data Availability Statement

The datasets presented in this study can be found in online repositories. The names of the repository/repositories and accession number(s) can be found here: NCBI BioProject PRJNA701067, https://www.ncbi.nlm.nih.gov/bioproject/PRJNA 701067.

## Ethics Statement

The animal study was reviewed and approved by Animal Experimentation Ethics Committee, School of Medicine, Southeast University. Written informed consent was obtained from the owners for the participation of their animals in this study.

## Author Contributions

YR, MN, and MC designed the experiments. MC, LX, GD, PL, and YZ performed the experiments. YL and MN performed the statistical analysis. MC and YR wrote the manuscript. All authors read and approved the final manuscript.

## Conflict of Interest

The authors declare that the research was conducted in the absence of any commercial or financial relationships that could be construed as a potential conflict of interest.
