# Supplementary material for: Inhibition of JAK-STAT Signaling Pathway Alleviates Age-Related Phenotypes in Tendon Stem/Progenitor Cells
Source: Front Cell Dev Biol. 2021 Mar 29;9:650250. doi: 10.3389/fcell.2021.650250 (PMC8039155; doi:10.3389/fcell.2021.650250)
Supplement: Supplementary file 1 [file Table_1.DOCX]

TABLE S1. Primer sequences for qRT-PCR.

| Target gene | Accession No. | Forward | Reverse | Amplicon (bp) |
| --- | --- | --- | --- | --- |
| p16^INK4A^ | NM_031550.1 | 5′-CCGATACAGGTGATGATGATGG-3′ | 5′-ACCGCAAATACCGCACGAC-3′ | 253 |
| IL6 | NM_012589.2 | 5′-AAGAGACTTCCAGCCAGTTGCC-3′ | 5′-TGTGGGTGGTATCCTCTGTGAAG-3′ | 107 |
| MMP9 | NM_031055.1 | 5′-GCAAACCCTGCGTATTTCCATT-3′ | 5′-GCGATAACCATCCGAGCGAC-3′ | 83 |
| CXCL12 | NM_001033882.1 | 5′- ATGTCGCCAGAGCCAACGT-3′ | 5′- GGATCCACTTTAATTTCGGGTCA-3′ | 126 |
| Egfr | NM_031507.1 | 5′-ATCAAAGTTCTGGGTTCAGGAGC-3′ | 5′-GACAGTGGAGGTCAGACAGATGC-3′ | 216 |
| Ar | NM_012502.1 | 5′-CTGATTCCTTTGCTGCCTTGT-3′ | 5′-ATTAGTGAAGGACCGCCAACC-3′ | 188 |
| IL6ST | NM_001008725.3 | 5′-TGAAGTCAGAGTGGGCAACAGA-3′ | 5′-GGTGGGCTGGGTTTCACTTTAT-3′ | 193 |
| JunD | NM_138875.4 | 5′-CAGTACGCAGTTCCTCTACCCTAAG-3′ | 5′-AACTGCTCAGGTTGGCGTAGA-3′ | 212 |
| Fos | NM_022197.2 | 5′-ACGCTCCAAGCGGAGACAGA-3′ | 5′-TCAAGTCCAGGGAGGTCACAGA-3′ | 181 |
| Bcl2 | NM_016993.1 | 5′-TTGTGGCCTTCTTTGAGTTCG-3′ | 5′-GCATCCCAGCCTCCGTTAT-3′ | 151 |
| Pim1 | NM_017034.1 | 5′-GCGGCGAACTCAAACTCATC-3′ | 5′-CTCAGGGACAGGCACCATCTA-3′ | 286 |
| Myc | NM_012603.2 | 5′-AAAACCCGACAGTCACGACG-3′ | 5′-GTAGCGACCGCAACATAGGAC-3′ | 256 |
| JAK2 | NM_031514.1 | 5′-CAGCAAACTAAAGAAGGCAGGA-3′ | 5′-TTCTCGCTCAACGGCAAAG-3′ | 103 |
| STAT3 | NM_012747.2 | 5′-TTTAACATTCTGGGCACGAACA-3′ | 5′-TGACAATCAAGGAGGCATCAC-3′ | 147 |
| IL1B | NM_031512.2 | 5′-TGACCTGTTCTTTGAGGCTGAC-3′ | 5′-CATCATCCCACGAGTCACAGAG-3′ | 272 |
| MMP3 | NM_133523.3 | 5′-CATGAACTTGGCCACTCCCT-3′ | 5′-TGGGTACCACGAGGACATCA-3′ | 178 |
| Nanog | NM_001100781.1 | 5′-AGGATAGGTTTCAGAGGCAAAGG-3′ | 5′-TCTGCCACCTCTTGCACTTCAT-3′ | 132 |
| Oct-4 | NM_001009178.2 | 5′-CTGTGGAGGGATGGCATACTG-3′ | 5′-GGCAAACTGCTCTAGCTCCTTC-3′ | 238 |
| Sca-1 | NM_012726.2 | 5′-AGGGCTGGTCATCCTGCTGT-3′ | 5′-TTTGGTGAGGAAGGGTGCTG-3′ | 317 |
| Ssea-1 | NM_022219.3 | 5′-GGCGTTTGAGAACTCACAGCA-3′ | 5′-GGTCCAGAAAGAGCAGGTAGGC-3′ | 197 |
| Tnmd | NM_022290.1 | 5′-GACCTATGGCATGGAGCACAC-3′ | 5′- TGTTTCATCGGTGCCATTTCC-3′ | 118 |
| Scx | NM_001130508.1 | 5′ -CGAGAACACCCAGCCCAAAC-3′ | 5′-CGTCTTTCTGTCACGGTCTTTG-3 | 82 |
| COL1A1 | NM_053304.1 | 5′-AGAGGCATAAAGGGTCATCGTG-3′ | 5′-AGACCGTTGAGTCCATCTTTGC-3′ | 161 |
| Nestin | NM_001308239.1 | 5′-TGGAGCAGGAGAAGCAAGGTC-3′ | 5′-CAAGGGGGAAGGGAAGGATGT-3′ | 281 |
| Dcn | NM_024129.1 | 5′-ATGGCAGTCTGGCTAATGTTCC-3′ | 5′-TGGTATGAAGGGAGGCAGAAGT-3′ | 169 |
| β-actin | NM_031144 | 5′-TGCTATGTTGCCCTAGACTTCG-3′ | 5′- GTTGGCATAGAGGTCTTTACGG-3′ | 240 |
